# Effects of TNF-α and IL-10-819 T>C single nucleotide polymorphisms on urogenital schistosomiasis in preschool children in Zimbabwe

**DOI:** 10.4102/ajlm.v10i1.1138

**Published:** 2021-04-29

**Authors:** Amos Marume, Theresa Chimponda, Arthur Vengesai, Caroline Mushayi, Jaclyn Mann, Takafira Mduluza

**Affiliations:** 1 Department of Infection Prevention and Control, School of Laboratory Medicine and Medical Sciences, College of Health Sciences, University of KwaZulu-Natal, Durban, South Africa; 2 Paraclinical Department, Faculty of Veterinary Sciences, University of Zimbabwe, Harare, Zimbabwe; 3 Department of Biochemistry, Faculty of Science, University of Zimbabwe, Harare, Zimbabwe

**Keywords:** *Schistosoma haematobium*, polymorphisms, cytokines, susceptibility, protective immunity

## Abstract

**Background:**

Knowledge gaps exist between host genetic factors and susceptibility to schistosomiasis.

**Objective:**

This study determined cytokine levels and single nucleotide polymorphisms of tumour necrosis factor (TNF)-α (rs1800629) and interleukin (IL)-10 (rs1800871) and their possible impact on susceptibility to schistosomiasis in preschool-age children in the Madziva area of Shamva district, Mashonaland Central province, Zimbabwe.

**Methods:**

Urogenital schistosomiasis was diagnosed using the urine filtration method, while a sandwich enzyme-linked immunosorbent assay was used for cytokine level determination. The survey was done in August 2015 and reinfection levels post treatment were assessed at 3, 6 and 12 months. Amplification refractory mutation system polymerase chain reaction with visualisation on 2% agarose gel electrophoresis was used for genotyping.

**Results:**

Schistosomiasis prevalence was found to be 10.5% (59/563). Reinfections were detected in only six children at 3 months and only one was reinfected at 12 months. There were no significant differences in TNF-α-308 G/A allele or genotype frequencies between the *Schistosoma haematobium* infected participants (*p* = 0.360) and uninfected participants (*p* = 0.279). However, no children with the IL-10-819 TT genotype had schistosomiasis. The TNF-α GG genotype corresponded with significantly lower TNF-α levels when compared with the GA or AA genotypes (*p* < 0.001), and TNF-α levels were significantly lower in infected children compared to uninfected children (*p* < 0.001).

**Conclusion:**

Higher TNF-α levels and lower IL-10 levels are potentially protective against schistosomiasis infection. The IL-10-819 TT genotype is potentially protective against infection through its association with lower IL-10 levels.

## Introduction

Preschool children from poor and endemic regions like sub-Saharan Africa are at high risk of schistosomiasis due to poor hygiene and sanitation, conditions that facilitate *Schistosoma* thriving. In these endemic regions, people living in both rural and urban centres use open water sources infested with intermediate snails harbouring the human host infectious stage of *Schistosoma*.^1^ Most of the people who are at risk of schistosomiasis worldwide have low awareness levels of the disease.^2,3^

Urogenital schistosomiasis has been described as a significant public health threat for preschool-age children (≤ 5 years); often they have higher infection burdens, and show serious morbidity and pathology relative to school-age children.^4^ Thus preschool children in endemic areas are regarded as a high-risk group needing inclusion in mass drug administration programmes.^1^ Praziquantel treatment, despite some few known side effects, is acceptable, safe and effective in all age ranges; treatment leads to 91% reduction in prevalence as well as 93% decrease in infection intensity.^4^ Praziquantel is known to remove the immunosuppressive effects of adult worms, and leads to the expression of worm antigens as a result of dying worm degeneration.^5^ Thus, praziquantel aids in immunity development.

Most endemic regions of Zimbabwe have prevalence rates that range from moderate to high risk according to World Health Organization guidelines.^6,7^ This qualifies those regions for mass praziquantel administration at least once every two years.^7^ Morbidity and mortality of schistosomiasis is well recognised to be a consequence of many host immune factors.^5^ People in endemic areas are known to develop protective immunity against reinfection, which offers hope for possible vaccines.^4^ The immunology of schistosomiasis is therefore of great significance in the management, control and possible eradication of infection.

Research, control strategies and treatment guidelines of schistosomiasis in children under five years of age are reported to have lagged behind those in other age groups.^5^ Significant knowledge gaps exist for the preschool-age group in relation to prevalence, morbidity, mortality, immunology, immunopathology, treatment effectiveness and safety, and impact of host genetic factors.

Tumour necrosis factor (TFN)-α and interleukin (IL)-10 have been associated with susceptibility and other key immune responses in many diseases including those caused by helminthes.^8,9,10^ We therefore assessed cytokine levels as well as cytokine genotypes in preschool children at risk of schistosomiasis.

## Methods

### Ethical considerations

The study was registered and ethically approved by Zimbabwe’s ethics board for bio-medical research (the Medical Research Council of Zimbabwe – MRCZ/A/1710). In addition, community leaders, the provincial medical director and the district medical officer also granted permission to conduct the study in Madziva, Shamva. The children agreed to participate in the study and their parents or guardians signed informed consent forms allowing their participation. The children and parents were educated on the aims, risks and benefits of the study. Participation was voluntary and participants were free to withdraw from the study at any time.

### Study population and sampling

The survey of urogenital schistosomiasis prevalence was conducted among 563 children aged between one year and five years through collection of urine specimens. Genotyping was done on 268 children who were willing and able to provide adequate blood. Convenience sampling was used, and the sample was several times larger than the 50 children per centre estimate used in other studies.^6^ The children were permanent residents of the Madziva area in Shamva district, Mashonaland Central province, Zimbabwe. The area was selected for this study based on its high annual rainfall, a condition conducive for the *Schistosoma* species and which could lead to high schistosomiasis endemicity.^6^ The study was a cross-sectional study, which was part of an ongoing longitudinal study on urogenital schistosomiasis for the 1–5 year age group (see funding section below). Participants infected with *Schistosoma mansoni* were excluded from this study.^11^ The survey was done in August 2015 and reinfection levels post treatment were assessed at 3, 6 and 12 months.

### Detection of *Schistosoma haematobium*

*Schistosoma haematobium* was diagnosed by the microscopic examination of urine specimens for the presence of parasite eggs using the urine filtration technique.^12^ In brief, urine specimens were collected from willing participants for three consecutive days. For diagnosis, 10 mL of urine was filtered through a nitrile filter membrane. The membrane was stained with iodine and examined under a light microscope. The same procedure was repeated on three consecutive days in order to prevent misdiagnosis due to day-to-day variation of egg excretion.^13^ If positive, the number of eggs in the entire sample of 10 mL of urine was counted and recorded as light infection if 1–10, moderate infection if 11–49 or heavy infection if more than 50 eggs. Participants who were *S. haematobium* positive were treated with a single dose of praziquantel (40 mg per kilogram of body weight). Bread and orange juice were given as supplementary food in order to enhance absorption as well as to alleviate the nauseating effects of praziquantel. Stool samples were collected once for *S. mansoni* diagnosis. Urine collection procedures and the diagnosis were repeated at 3, 6 and 12 months to assess reinfection levels post treatment with praziquantel.

### Blood sample collection

Approximately 5mL of blood was collected into ethylenediamine tetraacetic acid tubes. The tubes were centrifuged at 3000 revolutions per minute for 10 min using a ROTOFIX centrifuge. Plasma was collected and used to measure the levels of systemic cytokines. Host DNA was extracted from whole blood for genotyping using the Qiagen FlexiGene DNA extraction kit (Qiagen FlexiGene® DNA Handbook, Qiagen, Hilden, Germany).

### Genotyping

The TNF-α (rs18006290) and IL-10-819 T>C (rs1800871) polymorphisms were determined using amplification refractory mutation system polymerase chain reaction. The primers used were from Inqaba Biotechnology (Pretoria, South Africa) (Table 1). Primers specific for wild type genotype (1 *µ*L) and mutant genotype (1 *µ*L) were separately mixed with 1 *µ*L (10 mM) generic primer, 0.5 *µ*L (10 mM) forward and 0.5 *µ*L (10 mM) reverse internal control primers (human growth hormone), 12.5 *µ*L quick load Taq 2x master mix (New England Biolabs, Ipswich, Massachusetts, United States) and 4.5 *µ*L of sterile, nuclease-free water (New England Biolabs). Five *µ*L of the template DNA was added to the master mixes and loaded onto a PXE 0.2 thermocycler (Thermo Electron Corporation, Waltham, Massachusetts, United States). The forward and reverse internal control primer pair sequences were 5’-GCCTTCCCAACCATTCCCTTA-3’ and 5’- TCACGGATTTCTGTTGTGTTTC -3’. IL-10-819 T>C alleles were amplified using the following conditions: 1 min denaturation step at 95 °C; 10 cycles of 15 s at 95 °C, 50 s at 65 °C, and 40 s at 72 °C; and 20 cycles of 20 s at 95 °C, 50 s at 59 °C and 30 s at 72 °C, followed by cooling at 4 °C. TNF-α polymorphisms were amplified using the following conditions: 1 min denaturation step at 95 °C; 10 cycles of 15 s at 95 °C, 45 s at 60 °C, and 35 s at 72 °C; 25 cycles of 20 s at 95 °C, 45 s at 60 °C, and 45 s at 72 °C, followed by cooling at 4 °C. Amplification refractory mutation system polymerase chain reaction amplicons were analysed by electrophoresis on 2% agarose gel stained with ethidium bromide. The gels were viewed on an ultraviolet trans-illuminator to determine the presence or absence of the cytokine gene polymorphisms.

**TABLE 1:** Primers used in detecting TNF-α (rs18006290) and IL-10-819 T>C (rs1800871) polymorphisms among *S. haematobium* infected and uninfected preschool children from Shamva district, Mashonaland Central province, Zimbabwe, September 2018.

Cytokine	Position	Mutation	Primer sequence (5′-3′)	Fragment
rs1800871	−819	C→T	IL-10-819 C: CCC TTG TAC AGG TGA TGT AAC IL-10-819 T: CCC TTG TAC AGG TGA TGT AAT Generic: AGG ATG TGT TCC AGG CTC CT	233 base pairs
rs1800629	−308	G→A	TNF1: AGG TTT TGA GGG GCA TGG TNF2: AGG TTT TGA GGG GCA TGA Generic: CAG CGC AAA ACT TCC TTG GT	267 base pairs

Note: IL-10-819 C is the more common and ancestral allele, whereas IL-10-819 T is the less common or alternative allele of rs1800871. TNF1 is the more common and ancestral allele, whereas TNF2 is the less common or alternative allele of rs1800629.

### TNF-α and IL-10 determination

The levels of the cytokines were measured by an indirect enzyme-linked immunosorbent assay using Mabtech 3510-1H-6 kits (Mabtech AB, Büro Deutschland, Germany) according to the manufacturer’s instructions. The assay was performed in duplicate, and the data were averaged.

### Statistical analysis

Genotype frequencies of the TNF-α (rs1800629) and IL-10-819 T>C (rs1800871) between *S. haematobium* infected and uninfected participants were compared by a chi-square test. The Hardy-Weinberg equilibrium was assessed for TNF-α (rs1800629) and IL-10-819 T>C (rs1800871) *S. haematobium* infected and uninfected participants groups by chi-square. Genotype data were analysed with the software International Business Machines Corporation Statistical Package for Social Sciences for Windows, version 19.0. (International Business Machines Corporation, Armonk, New York, United States). Unpaired *t*-tests were used to compare cytokine levels between infected and uninfected participants and between different genotypes, and analysis of variance was used to compare cytokine levels between light, moderate and heavy infection groups. *p*-values less than 0.05 were considered statistically significant.

## Results

### Prevalence of *S. haematobium* infection

There was no significant difference (*p* = 0.823) between boys (*n* = 294) and girls (*n* = 269) recruited. The prevalence of *S. haematobium* infection was 10.5% (59/563), with an even distribution between boys (50.8%) and girls (49.2%) (*p* = 0.82).

### TNF-α and IL-10 genotypes are not associated with infection status or intensity

All genotypes of rs1800629 and rs1800871 were represented in the population and, consistent with previous literature, the TNF-α-308 GA genotype and IL-10 CT genotype were the most common. None of the genotypes studied showed significant differences between the infected and uninfected groups (Table 2). It was found that the rs1800629 single nucleotide polymorphism was in line with the Hardy-Weinberg equilibrium in uninfected participants. However, the rs1800871 single nucleotide polymorphism was not in line with the Hardy-Weinberg equilibrium in both *S. haematobium* infected and uninfected participants.

**TABLE 2:** Genotype distributions of TNF-α (rs1800629) and IL-10-819 T>C (rs1800871) between *S. haematobium* infected and uninfected preschool children from Madziwa, Shamva district, Zimbabwe, September 2018.

SNPs	Uninfected		Infected		*X*^2^	*p*	Uninfected		Infected	
	*n*	%	*n*	%			*X*^2^ for HWE	*p*-value for HWE	*X*^2^ for HWE	*p*-value for HWE
rs1800629	-	-	-	-	2.276	0.320	1595.664	*p* < 0.001	70.311	*p* < 0.001
GG	21	8.7	3	12.5	-	-	-	-	-	-
AG	158	65.3	18	75	-	-	-	-	-	-
AA	63	26.0	3	12.5	-	-	-	-	-	-
rs1800871	-	-	-	-	0.951	0.622	6.781	0.034	-	-
CC	12	24.5	2	40.0	-	-	-	-	-	-
CT	32	65.3	3	60.0	-	-	-	-	-	-
TT	5	10.2	0	0.0	-	-	-	-	-	-

Note: Tests were done on 268 preschool children; however, blood samples obtained from some participants were not sufficient to carry out both genotyping tests, thus the rs1800629 or rs1800871 alleles could not be determined in those participants. Because rs1800871 SNP was not in line with HWE, there are no *p*-values reported.

SNP, single nucleotide polymorphism; HWE, Hardy-Weinberg equilibrium.

When the data were stratified by infection intensities, there was no significant difference between the infected and uninfected groups (Table 3). Interestingly, 10 children had the IL-10 TT genotype (associated with low production of IL-10) and none of them were infected, raising the hypothesis that the TT genotype might be protective against *S. haematobium*, although this was not statistically significant.

**TABLE 3:** Genotype distributions of TNF-α (rs1800629) and IL-10-819 T>C (rs1800871) grouped according to *S. haematobium* infection intensity in preschool children from Madziwa, Shamva district, Zimbabwe, September 2018.

Single nucleotide polymorphisms	No infection		Light infection		Moderate infection		Heavy infection		*X*^2^	*p*
	*n*	%	*n*	%	*n*	%	*n*	%		
rs1800629	-	-	-	-	-	-	-	-	2.847	0.828
GG	21	8.7	2	11.8	1	16.7	0	0.0	-	-
AG	158	65.3	13	76.5	4	66.7	1	100	-	-
AA	63	26.0	2	11.8	1	16.7	0	0.0	-	-
rs1800871	-	-	-	-	-	-	-	-	1.131	0.889
CC	12	24.5	1	33.3	1	50.0	0	0.0	-	-
CT	32	65.3	2	66.7	1	50.0	0	0.0	-	-
TT	5	10.2	0	0.0	0	0.0	0	0.0	-	-

Note: Light = 1-10, moderate = 11-49 and heavy infection = 50 and above schistosome eggs per 10 mL of urine.

### Cytokine levels were associated with cytokine genotypes and infection

**FIGURE 1:**
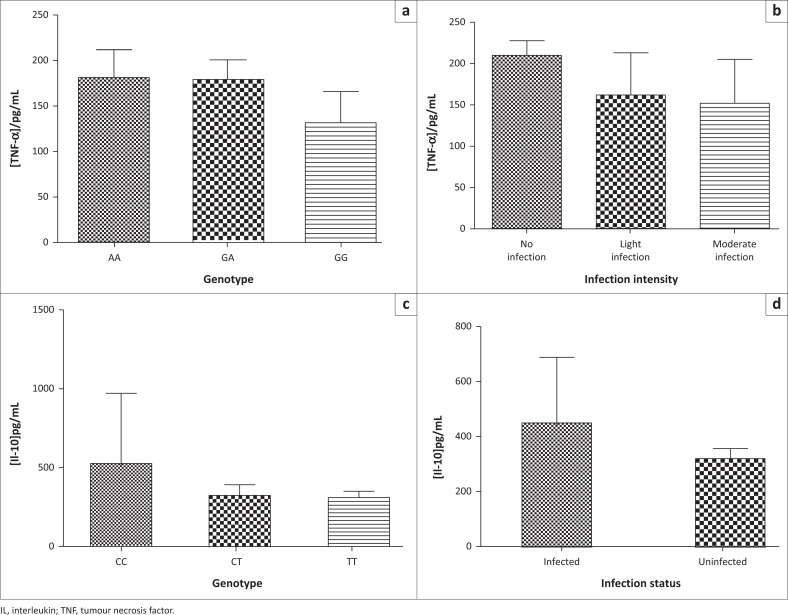
Genotypes, cytokine levels and infection status in preschool children from Madziwa, Shamva district, Zimbabwe, September 2018. (a) Genotypes and TNF-α production (mean level for AA is 179.3 ± 31.2 pg/mL *n* = 34, GA is 176.8 ± 23.8 pg/mL *n* = 98 and GG is 131.6 ± 36 pg/mL *n* = 14); (b) TNF-α levels against infection intensity (239 ± 71.6 pg/mL *n* = 23 and 210 ± 17.6 pg/mL *n* = 286); (c) Genotypes against IL-10 plasma levels (CC – 528.5 pg/mL, CT – 323.8 pg/mL and TT – 309.4 pg/mL) (d) IL-10 plasma levels against infection status (452.5 pg/mL and 376.4 pg/mL).

The TNF-α GG genotype corresponded with significantly lower TNF-α levels when compared with the GA or AA genotypes (Figure 1a) (*p* < 0.001), and TNF-α levels were significantly lower in infected children compared to uninfected children (Figure 1b). The IL-10 TT genotype corresponded with significantly lower IL-10 levels when compared with CC (Figure 1c). IL-10 levels were significantly lower in uninfected children compared to infected children (Figure 1d) (unpaired t-test; *p* < 0.001).

### Reinfection

Reinfections at three months post treatment were detected in 10.2% (6/59) of children who were previously infected. At six months there were no reinfections observed and only one child was reinfected after one year post treatment.

## Discussion

The study describes the prevalence of urogenital schistosomiasis in preschool children and the impact of single nucleotide polymorphisms of TNF-α-308 G/A and IL-10-819 T>C gene promoter regions on cytokine levels, susceptibility to *S. haematobium* infections and treatment outcomes. The results highlight and support existing evidence on schistosomiasis prevalence levels (10.5%) in children aged between one and five years and also support the possible effectiveness of mass drug administrations and immune development facilitation effects of praziquantel treatment as only six were reinfected one year post treatment. The study showed that cytokine levels were different depending on the individual’s genotype: the IL-10-819 CC genotype corresponded with the highest levels of IL-10, while the CT and TT genotypes corresponded with moderate and low levels of IL-10, and the TNF-α-308 AA genotype corresponded with the highest, GA with moderate to high and GG with low TNF-α levels. A major finding was that TNF-α and IL-10 levels might predict susceptibility to *S. haematobium* infection, with higher TNF-α and lower IL-10 levels appearing to confer protection. Interestingly, all 10 children who had the IL-10 TT genotype (associated with low production of IL-10) were uninfected, suggesting that this genotype may confer a protective effect, although this was not supported by statistical tests. Higher levels of IL-10 have also previously been linked with possible *S. haematobium* susceptibility.^14^ IL-10 has immunomodulatory effects which may explain the susceptibility of individuals with high IL-10 levels to schistosome infections. Higher levels of IL-10 have also been demonstrated to be protective against serious morbidity.^14,15^ Our study suggests that higher levels of the pro-inflammatory cytokine (TNF-α) could aid in immunity development against schistosomes and that the intensity of infection was somewhat linked to TNF-α levels as those with moderate levels of TNF-α had a light infection (1–10 eggs/10 mL of urine) and those with low levels had moderate infection (11–49 eggs/10 mL), although this was not statistically significant. This supports the hypothesis that TNF-α is also helpful in limiting the proliferation of the parasite.

The allele or genotype frequencies were as expected in Africans: IL-10 819 CT genotype frequencies were above 50% and TNF-α-308 GA genotype frequencies were above 80%.^16,17^ The relationship between cytokine levels and genotypes was also consistent with that in previous studies.^18^ IL-10-819 CC genotype corresponded to high levels of plasma IL-10, CT to moderate, and TT to low IL-10 levels. For TNF-α-308, AA genotype corresponded to high, GA corresponded to moderate to high and GG corresponded to low plasma levels of TNF-α.^18^ IL-10 promoter SNPs, –1082G/A, –819C/T and –592C/A, are involved in differential IL-10 gene transcription and hence differential IL-10 production.^18^ The findings highlighted a general lack of association between any genotype and urogenital schistosomiasis; however, the data pointed to a possible protective effect of IL-10-819 TT. Given that actual cytokine levels, rather than the SNPs studied here, show an association with susceptibility to *S. haematobium* infection, the findings highlight complexities that may exist in the expression of those genes and that additional SNPs may play an important role in the expression of these cytokines. Although no significant association was demonstrated in this study, IL-10 haplotypes GCC and GTA were previously linked to high to moderate IL-10 production and were demonstrated to be associated with schistosomiasis.^18^ This may highlight the need for a larger sample size to demonstrate the suggested link between genotypes and susceptibility or the need to identify and study other polymorphisms that affect expression of IL-10 and TNF-α.

Peripheral blood mononuclear cells from subjects with schistosomiasis have been shown to produce eightfold greater levels of egg antigen-driven TNF-α, leading to a 99-fold greater mean TNF-α to IL-10 ratio, relative to negative controls.^15^ Some studies have established a positive relationship between infection intensity and TNF-α production.^19,20^ IL-10 elevation in schistosome infections has also been demonstrated, with some authors suggesting both TNF-α and IL-10 as possible biomarkers in schistosomiasis.^19,20,21^ Thus the high levels of IL-10 in infected individuals could have been a result of the infection rather than the cause of infection. However, there are previous reports that high levels of IL-10 predispose to reinfection^8,9,10^ and we found that no individuals with the IL-10 TT genotype (which was also associated with lower IL-10 levels) were infected. Taken together, these observations support the hypothesis that the association between IL-10 levels and schistosomiasis in our study is due to higher IL-10 levels predisposing to infection and lower levels conferring protection. Some studies detected elevated levels of TNF-α in infected individuals relative to uninfected controls especially in acute schistosomiasis.^22^ The findings of this study however contradict those observations as low levels of TNF-α were associated with infection. According to the findings of this study, higher levels of TNF-α and lower levels of IL-10 appear to be protective against *S. haematobium*.

In this study most children did not get reinfected after 3, 6 and 12 months, suggesting the possible efficacy of praziquantel mass drug administrations in elimination of schistosomiasis. The few that were reinfected fall into the category of moderate to high production of TNF-α (individuals with the TNF-α GA and AA genotypes), which appears contradictory to our observation that high levels of TNF-α are negatively associated with infection. The effects of praziquantel treatment on immunological profiles and immunity to reinfection were reported to be similar in all age groups of children.^4^ However, TNF-α was shown to play a complex role in propagating the intramammalian stages of the schistosome life cycle – an effect that helps schistosomes survive in the host.^23^ This might explain the observed few reinfections. Alternatively, some of the ‘reinfections’ might represent treatment failure cases, particularly since 5 of the 6 cases were observed at three months post treatment. Some studies suggest that IL-10 blocks the development of immunity after praziquantel treatment, thus reinfection is better prevented when praziquantel treatment is administered in individuals with low or blocked IL-10.^24^ The reinfections could also be explained by the fact that the participants were inhabitants of an endemic area, and that praziquantel administrations were not coupled with other key interventions like killing of intermediate hosts and provision of safe water. Thus, the study could not conclusively explain the reinfections. The low reinfection levels over the 12-month period could help support sustained community-based praziquantel administrations. Such efforts can be reasonably expected to significantly reduce the schistosome burdens in endemic areas.

The prevalence of 10.5%, although still high, may suggest effectiveness of prior mass drug administration and health education efforts as it is below 20.8% (nationally reported urogenital schistosomiasis prevalence in 2012).^25^ In 2011, the province (Mashonaland Central) had an overall prevalence of 26.1% (23.46–28.90) and Shamva was also categorised among the top districts with heavy intensities (53.2%) of *S. haematobium* infection.^6^ More efforts like snail control, preventative chemotherapy and improvement of hygiene and sanitation are still required as these have at some point sustained prevalence below 10% in areas like Hippo Valley Sugar Estates, Zimbabwe.^26^ The observed lack of differences in prevalence of schistosomiasis in boys and girls is in contrast to what was established by major surveys conducted in Zimbabwe previously.^6,27^ In those primary school surveys, girls consistently had a lower prevalence of infection compared to their male counterparts. The absence of differences between boys and girls can be attributed to many, often overlapping, activities or chores like swimming, washing and fetching water from unprotected sources. The major factor is also that the water contact of preschool children is parent or guardian related, so no differences could be expected. Gender differences in activities often start to vary at around age six, thus similar prevalence of infection is expected in children under six years of age and gender differences may emerge thereafter.^28^

### Limitations

The prevalence of schistosomiasis was low and could have limited the ability to highlight an association of any of the studied genotypes with susceptibility to *S. haematobium*. Although the sample was larger than the recommended 50 participants per centre, resources limited the ability to have more participants which could have helped in establishing the rare phenomenon. The results could also have been affected by the fact that we had a few individuals who had heavy infection intensities. Some of the recruited children were not willing or able to provide adequate blood for genotyping. Future studies are recommended to include many cytokines and all possible SNPs known to influence cytokine gene expression.

### Conclusion

Prevalence of urogenital schistosomiasis among boys and girls was indistinguishable statistically. GA was the most frequent TNF-α-308 genotype. AA, GA and GG genotypes were associated with a high, moderate to high and low production of TNF-α, respectively. TNF-α-308 G/A and IL-10-819 T>C polymorphisms were not significantly associated with susceptibility to *S. haematobium* infection, although no individuals with the IL-10 TT genotype (*n* = 10), which is associated with lower IL-10 levels, were infected in this study. Higher TNF-α and lower IL-10 levels were negatively associated with infection and may confer protection against schistosomiasis. Praziquantel helped in reducing reinfection levels of schistosomiasis in preschool children staying in an endemic area.

The findings of this study highlight the need for continued efforts (mass drug administrations, better sanitation and hygiene and health education and promotion) to reduce the prevalence of schistosomiasis to zero among children in endemic areas of Zimbabwe. Additionally, there is the need for immune-based interventions that encourage TNF-α but limit IL-10 production as this will promote immunity against *S. haematobium*.
